# *Curcumin longa* extract-loaded nanoemulsion improves the survival of endotoxemic mice by inhibiting nitric oxide-dependent HMGB1 release

**DOI:** 10.7717/peerj.3808

**Published:** 2017-09-14

**Authors:** Min Young Ahn, Jung Seok Hwang, Su Bi Lee, Sun Ah Ham, Jinwoo Hur, Jun Tae Kim, Han Geuk Seo

**Affiliations:** 1 Department of Food Science and Biotechnology of Animal Products, Konkuk University, Seoul, South Korea; 2 Department of Food Science and Technology, Keimyung University, Daegu, South Korea

**Keywords:** Nanoemulsion, High mobility group box 1, Curcuma longa, Endotoxemia

## Abstract

**Background:**

High mobility group box 1 (HMGB1) is a well-known damage-related alarmin that participates in cellular inflammatory responses. However, the mechanisms leading to HMGB1 release in inflammatory conditions and the therapeutic agents that could prevent it remain poorly understood. This study attempted to examine whether the *Curcumin longa* herb, which is known to have anti-inflammatory property, can modulate cellular inflammatory responses by regulating HMGB1 release.

**Methods:**

The murine macrophage RAW264.7 cells were treated with lipopolysaccharide (LPS) and/or a * C. longa* extract-loaded nanoemulsion (CLEN). The levels of released HMGB1, nitric oxide (NO) production, inducible NO synthase (iNOS) expression, and phosphorylation of mitogen-activated protein kinases were analyzed in RAW264.7 macrophages. The effects of CLEN on survival of endotoxemic model mice, circulating HMGB1 levels, and tissue iNOS expression were also evaluated.

**Results:**

We have shown that a nanoemulsion loaded with an extract from the *C. longa* rhizome regulates cellular inflammatory responses and LPS-induced systemic inflammation by suppressing the release of HMGB1 by macrophages. First, treatment of RAW264.7 macrophages with the nanoemulsion significantly attenuated their LPS-induced release of HMGB1: this effect was mediated by inhibiting c-Jun N-terminal kinase activation, which in turn suppressed the NO production and iNOS expression of the cells. The nanoemulsion did not affect LPS-induced p38 or extracellular signal-regulated kinase activation. Second, intraperitoneal administration of the nanoemulsion improved the survival rate of LPS-injected endotoxemic mice. This associated with marked reductions in circulating HMGB1 levels and tissue iNOS expression.

**Discussion:**

The present study shows for the first time the mechanism by which *C. longa* ameliorates sepsis, namely, by suppressing NO signaling and thereby inhibiting the release of the proinflammatory cytokine HMGB1. These observations suggest that identification of agents, including those in the herb *C. longa*, that can inhibit HMGB1 production and/or activity may aid the treatment of endotoxemia.

## Introduction

High mobility group box 1 (HMGB1) is a non-histone nuclear protein that participates in diverse biological processes that associate with genome integrity and gene regulation in the nucleus (Ueda & Yoshida, 2010). However, HMGB1 also has non-nuclear functions: when immune cells are exposed to pathogen-related molecules such as lipopolysaccharide (LPS), they secrete HMGB1 into the extracellular compartment, where it acts as a proinflammatory cytokine (Andersson & Tracey, 2011). In particular, macrophage-derived HMGB1 was found to mediate lethal LPS-induced sepsis in mice: this function occurred late after LPS administration (Wang et al., 1999). Moreover, high serum levels of HMGB1 in sepsis patients associate with death, tissue damage, and multiple organ failure (Wang et al., 1999; Yang et al., 2004; Sundén-Cullberg et al., 2005). Significantly, administration of HMGB1 inhibitors or antibodies specific for HMGB1 improves inflammation-related disorders such as sepsis, colitis, and ischemic reperfusion (Wang et al., 1999; Davé et al., 2009; Andrassy et al., 2008). More recently, we showed that rosiglitazone, a ligand for peroxisome proliferator-activated receptor (PPAR)γ, improves the survival of endotoxemic model mice by inhibiting the secretion of HMGB1 into the circulation (Hwang et al., 2012; Hwang et al., 2015). Furthermore, PPARγ-mediated induction of SIRT1 (sirtuin 1; silent mating type information regulation 2 homologue 1) expression inhibited the HMGB1 release by direct interaction with HMGB1 (Hwang et al., 2015). Accordingly, the blockade of HMGB1 secretion into extracellular fluid is a critical determinant in the relieving of septic conditions caused by inflammation.

*Curcuma longa* Linn is a perennial herb that belongs to the Zingiberaceae family. It is a common ingredient in many food and health supplements in Africa and South Asia, including India and China (Gupta et al., 2013). In particular, the powdered rhizome of *C. longa* (commonly known as turmeric) has anti-inflammatory, antioxidant, antibacterial, and chemopreventive activities (Gupta et al., 2013; Chandrasekaran et al., 2013; Kumar & Singh, 2008). These diverse biological activities of the *C. longa* rhizome are due to multiple compounds, including curcumin, alkaloids, polyphenols, and terpenoids (Gupta et al., 2013).

Multiple studies have revealed the anti-inflammatory activity of *C. longa.* For example, when carbon tetrachloride-intoxicated mice are fed an aqueous extract of *C. longa*, the immune functions of their peritoneal macrophages improve significantly (Sengupta, Sharma & Chakraborty, 2011). Moreover, when normal murine splenocytes are treated with the polysaccharide fraction of *C. longa* extract, their LPS-stimulated release of prostaglandin E2 and interleukin-12 is potently inhibited (Sengupta, Sharma & Chakraborty, 2011). Furthermore, when LPS-stimulated murine microglial BV-2 cells and inflamed human intestinal Caco-2 cells are treated with a curcumanoid and terpenoids from a *C. longa* extract, respectively, their inflammation improves markedly: this is achieved by regulation of the nitric oxide (NO) signaling pathway (Somchit et al., 2014; Xu et al., 2015). In addition, the ethyl acetate fraction of *C. longa* exhibits strong scavenging activity for peroxynitrite, which is a cytotoxic intermediate that is generated by a reaction between NO and superoxide anion (Kim et al., 2003).

Although these observations indicate that *C. longa* extracts or constituents have anti-inflammatory properties, the mechanisms by which they dampen inflammation remain poorly understood. In particular, it is unclear how *C. longa* protects animal models from endotoxemic responses. Consequently, the present study was performed to investigate the effect of a *C. longa* extract-loaded nanoemulsion (CLEN) on the LPS-induced release of HMGB1 by the murine macrophage line RAW264.7. We found that CLEN suppresses HMGB1 release by the LPS-stimulated RAW264.7 cells. Moreover, injection of endotoxemic model mice with CLEN markedly reduced their mortality rate. Notably, CLEN inhibited HMGB1 release by suppressing the c-Jun N-terminal kinase (JNK); this in turn blocked the NO signaling pathway and thereby inhibited the release of HMGB1 into the extracellular compartment.

## Materials & Methods

### Preparation of the *C. longa* extract-loaded nanoemulsion (CLEN)

The extract of *C. longa* was prepared by using 50% ethanol for 24 h. The concentrated extract contained 10.43 mg/mL curcumin. The oil phase was prepared by dissolving 173.92 g *C. longa* extract with 86.96 g MCT (medium chain triglyceride) oil containing 26.09 g soy lecithin. The aqueous phase was prepared by mixing 17.39 g Tween 80, 173.9 mL *C. longa* extract, and 869.57 mL distilled water. A coarse emulsion was prepared by magnetic stirring at room temperature for 2 h. Nanoemulsions were prepared by further homogenizing the coarse emulsion. For this, the emulsion was first subjected to high speed homogenization at 5,000 rpm for 10 min, then to ultrasonication with a Vibra Cell (VCX-750, Sonics & Materials, Inc., Sandy Hook, CT, USA) for 15 min, and finally to high pressure homogenization under 10,000 psi for three cycles. The CLEN powder was prepared by spray drying 1,000 mL CLEN and 113.06 g dextrin.

### Characterization of CLEN

The mean droplet size and polydispersity index (PDI) of CLEN were determined by dynamic light scattering using a particle size analyzer (ELSZ-1000; Otsuka Electronics Co., Ltd., Osaka, Japan). Thus, 1 mL CLEN was added to a polystyrene latex cell, and the mean droplet size and PDI were measured at 25 °C with a detector angle of 90° and wavelength of 633 nm. The curcumin content in CLEN was measured by UV-Vis spectrophotometry at 425 nm.

### Cell culture

Murine macrophage RAW264.7 cells were obtained from the Korean Cell Line Bank (Seoul, Korea). The cells were maintained in a humidified atmosphere of 5% CO_2_ at 37 °C in Dulbecco’s modified Eagle’s medium containing 10% heat-inactivated fetal calf serum, 100 U/ml penicillin, and 100 µg/ml streptomycin.

### MTT assay

RAW264.7 cells seeded into a 24-well plate were incubated with various concentrations of CLEN for 24 h, after which the MTT solution was added to the culture medium. After incubation for 4 h, the medium was removed, the crystal formazan was dissolved, and the absorbance at 570 nm was determined.

### Measurement of released HMGB1

The HMGB1 released into the culture medium was measured as described previously (Hwang et al., 2015). Briefly, RAW264.7 cells grown to sub-confluency were maintained in serum-free medium for 24 h and then incubated with or without LPS in the presence or absence of CLEN for 24 h. Aliquots of conditioned culture media from equal numbers of cells were precipitated with 80% cold acetone, after which the resulting pellets were resuspended in SDS-PAGE sample buffer. The levels of HMGB1 released into the culture medium were analyzed by immunoblot.

### Western blot analysis

The protein expression was measured by immunoblot analysis as described previously (Hwang et al., 2015). Briefly, RAW264.7 cells were incubated with the indicated reagents for the indicated time periods and then lysed in PRO-PREP Protein Extraction Solution (iNtRON Biotechnology, Seoul, Korea). Aliquots of whole-cell lysates or conditioned media were subjected to Western blot analysis with specific antibodies.

### Measurement of NO

NO formation was determined by spectrometry that measured the levels of nitrite (an oxidized form of NO) that had accumulated in the culture medium (Seo, Nishinaka & Yabe-Nishimura, 2000). After incubation for the indicated time period, 100 µl aliquot of conditioned culture medium was reacted with 100 µl of Griess reagent.

### Animal model of endotoxemia and the survival test

The Institutional Animal Care and Use Committee of Konkuk University approved all of the present animal studies (approval number: KU15140). Endotoxemic animal models were generated by intraperitoneally injecting male or female 7-week-old BALB/c mice (20–25 g) with bacterial endotoxin (10 mg/kg *E. coli* LPS 0111:B4) as described previously (Hwang et al., 2015). Briefly, mice were randomly assigned to the following four groups: vehicle (DMSO), 10 mg/kg LPS, 10 mg/kg LPS plus 50 mg/kg CLEN, 10 mg/kg LPS plus 50 mg/kg CLEN plus 2.5 mg/kg sodium nitroprusside (SNP), 10 mg/kg LPS plus 2.5 mg/kg SNP, 50 mg/kg CLEN, and/or 2.5 mg/kg SNP.

### Determination of serum HMGB1 levels and tissue iNOS expression

The HMGB1 levels in the serum of the septic mice described above were determined by collecting the blood 18 h after LPS injection and subjecting an aliquot of serum to immunoblot analysis of HMGB1. To measure iNOS expression, the tissues from the septic mice were ground under liquid nitrogen and lysed in PRO-PREP Protein Extraction Solution (iNtRON Biotechnology). Aliquots of the tissue lysates were then subjected to Western blot analysis to determine the iNOS expression levels.

### Real-time polymerase chain reaction (PCR)

The levels of each mRNA were analyzed by real-time PCR. Total RNA was extracted using TRIzol reagent (Invitrogen, Carlsbad, CA, USA) and reverse-transcribed into cDNA by a TOPscript RT DryMIX kit (Enzynomics, Seoul, Korea). Equal amounts of cDNA were amplified in a 10 µl reaction solution containing 1  × SYBR PCR master mix (Takara Bio Inc., Otsu, Japan) and 10 µM primers. Real-time PCR was carried out by a Rotor Gene RG-3000 (Corbett Life Science, Sydney, Australia) according to the PCR conditions as follows: initial denaturation for 5 min at 95 °C, followed by 35 cycles of 20 s at 95 °C, 50 s at 58 °C, and 40 s at 72 °C. The primers were *tumor necrosis factor* (*TNF)- α*, 5′-TCCCAAATGGCCTCCCTCTCA-3′ and 5′-GTGGTTTGCTACGACGTGG-3′; *interleukin (IL)-1 β*, 5′-AAACCTTTGACCTGGGCTGTCC-3′ and 5′-TGCCTGCCTGAAGCTCTT GTT-3′; *monocyte chemotactic protein (MCP)-1*, 5′-TGCAGTTAACGCCCCACTCAC-3′ and 5′-CAGCTTCTTTGGGACACCTGC-3′; *GAPDH*, 5′-CATGGCCTTCCGTGTTCCTA-3′ and 5′-CCTGCTTCACCACCTTCTTGAT-3′.

### Statistical analysis

The data are presented as mean ± standard error. Differences were assessed for significance by a one-way ANOVA followed by the Tukey–Kramer test (*p* < 0.05).

## Results

### Characterization of CLEN and the CLEN powder

**Figure 1:**
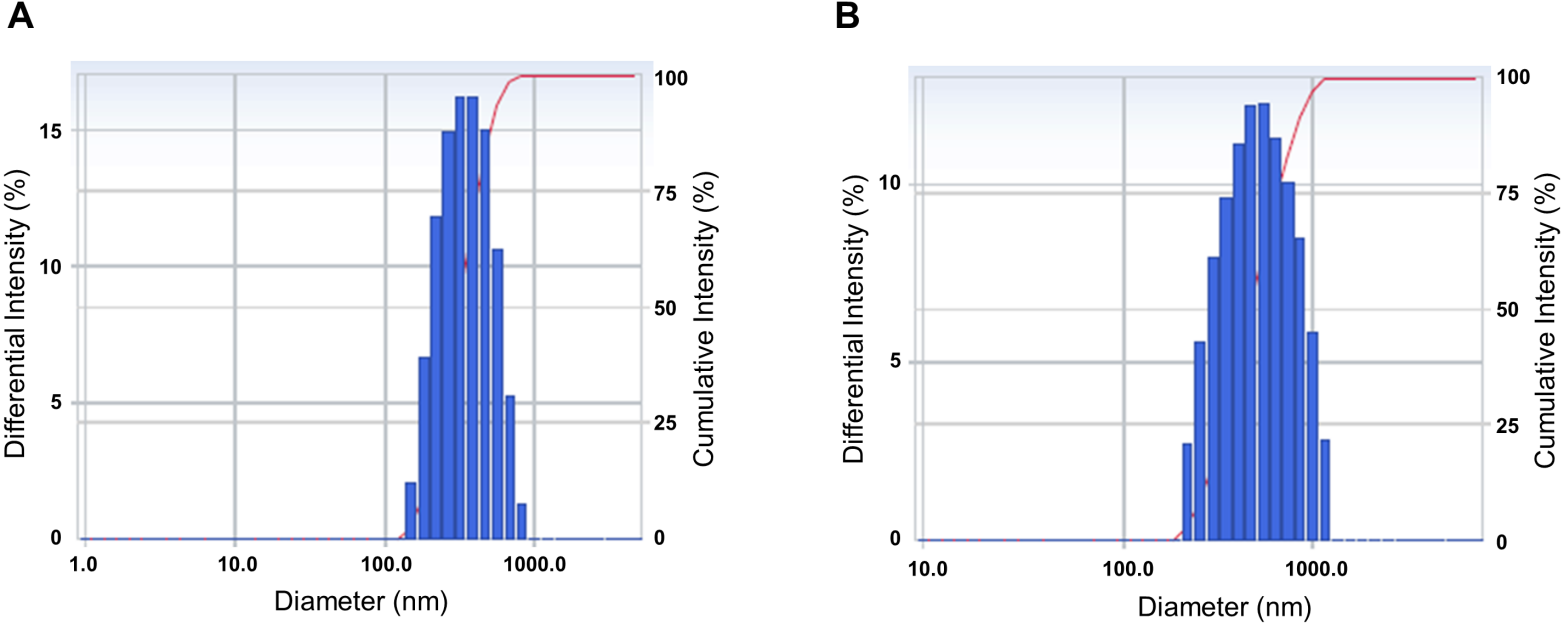
Characterization of the physical properties of the *Curcumin longa* extract-loaded nanoemulsion (CLEN) and the CLEN powder. To measure the physical properties, 1 mL CLEN (A) and the CLEN powder (B) was added to a polystyrene latex cell, and the mean droplet size and polydispersity index were measured at 25°C with a detector angle of 90°and a wavelength of 633 nm by using a zeta potential and particle size analyzer. Each sample was measured at least three times.

We analyzed the particle size and size distribution of the nanoemulsion and the nanoemulsion powder because these crucial variables indicate the stability, solubility, release rate, and bioavailability of nanoparticles for encapsulation (Tamjidi et al., 2013). As shown in Fig. 1, the size distribution of both CLEN and the CLEN powder showed a single spectrum. This indicates that the particle sizes of CLEN and the CLEN powder were uniformly distributed, although the distribution of the CLEN powder was broader than that of CLEN. The mean droplet size of CLEN and the CLEN powder were 323.6 nm and 492.2 nm, respectively.

### Effect of CLEN on LPS-stimulated release of HMGB1 by RAW264.7 cells

**Figure 2:**
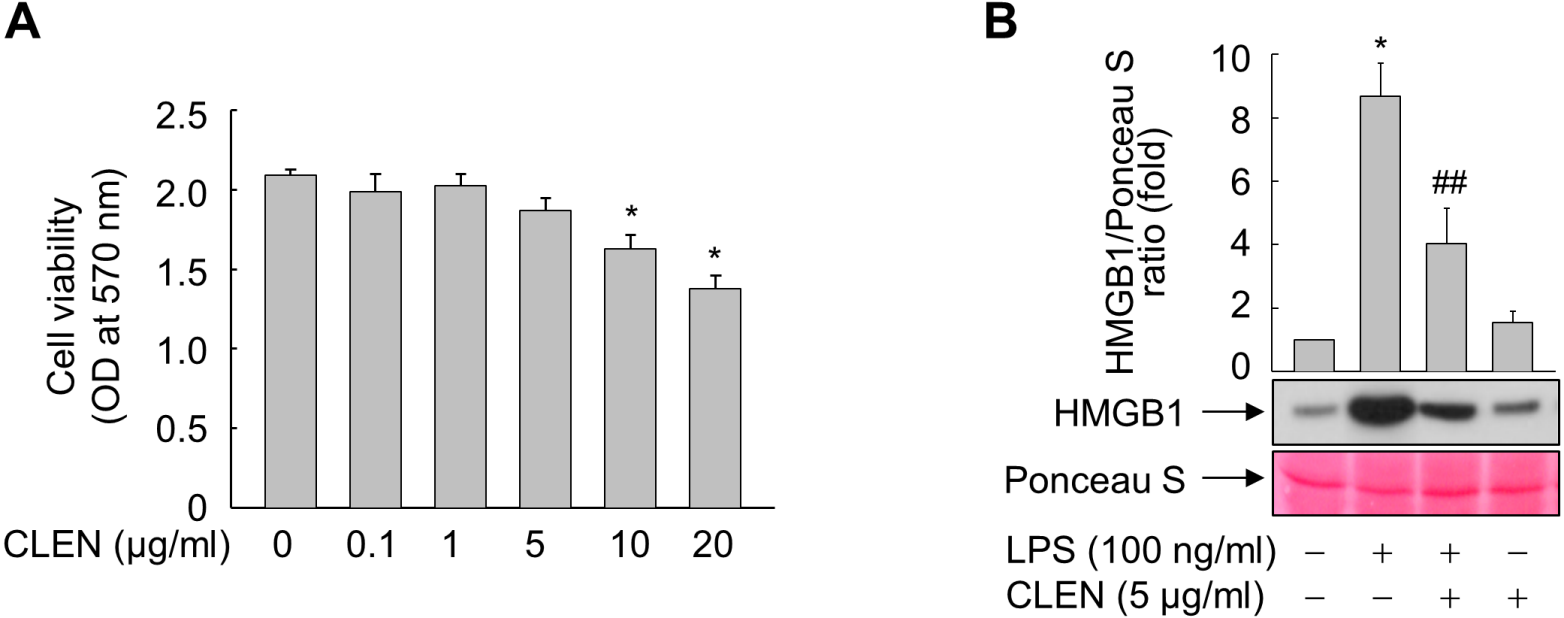
Effect of the *Curcumin longa* extract-loaded nanoemulsion (CLEN) on the lipopolysaccharide (LPS)-stimulated release of high-mobility group box 1 (HMGB1) by RAW264.7 cells. (A) RAW264.7 cells were incubated with the indicated concentrations of CLEN for 24 h and cell viability was assessed by using the MTT assay. (B) RAW264.7 cells grown to sub-confluency were maintained in serum-free medium for 24 h and then incubated for 24 h with or without LPS in the presence or absence of CLEN. Equal volumes of the conditioned media were subjected to immunoblot analysis with an anti-HMGB1 antibody. Ponceau S staining served as a loading control. A representative blot from three independent experiments is shown. The band intensities in the three experiments were quantitated by using an image analyzer. The mean ± S.E. ratios of HMGB1 to Ponceau S are plotted above the blot (*n* = 3). ^∗^*p* < 0.01 compared to the untreated cells; ^##^*p* < 0.05 compared to the cells that were treated with LPS alone.

To determine the optimal dosage, RAW264.7 cells were treated with 0, 0.1, 1.0, 5.0, 10, or 20 µg/ml of CLEN for 24 h. Although CLEN did not exhibited any cytotoxic effects in the concentrations below 5 µg/ml, the cell viability was significantly reduced in cells treated with 10 or 20 µg/ml CLEN (Fig. 2A). Thus, we chose the submaximal dosage of 5 µg/ml CLEN for most experiments on RAW264.7 cells.

We then assessed the anti-inflammatory activity of CLEN by culturing RAW264.7 cells with LPS in the presence and absence of CLEN and then examining their release of HMGB1. LPS increased the release of HMGB1 into the extracellular milieu and this effect was significantly suppressed by treatment with CLEN (Fig. 2B).

### Effect of CLEN on the NO production and iNOS expression of LPS-treated RAW264.7 cells

**Figure 3:**
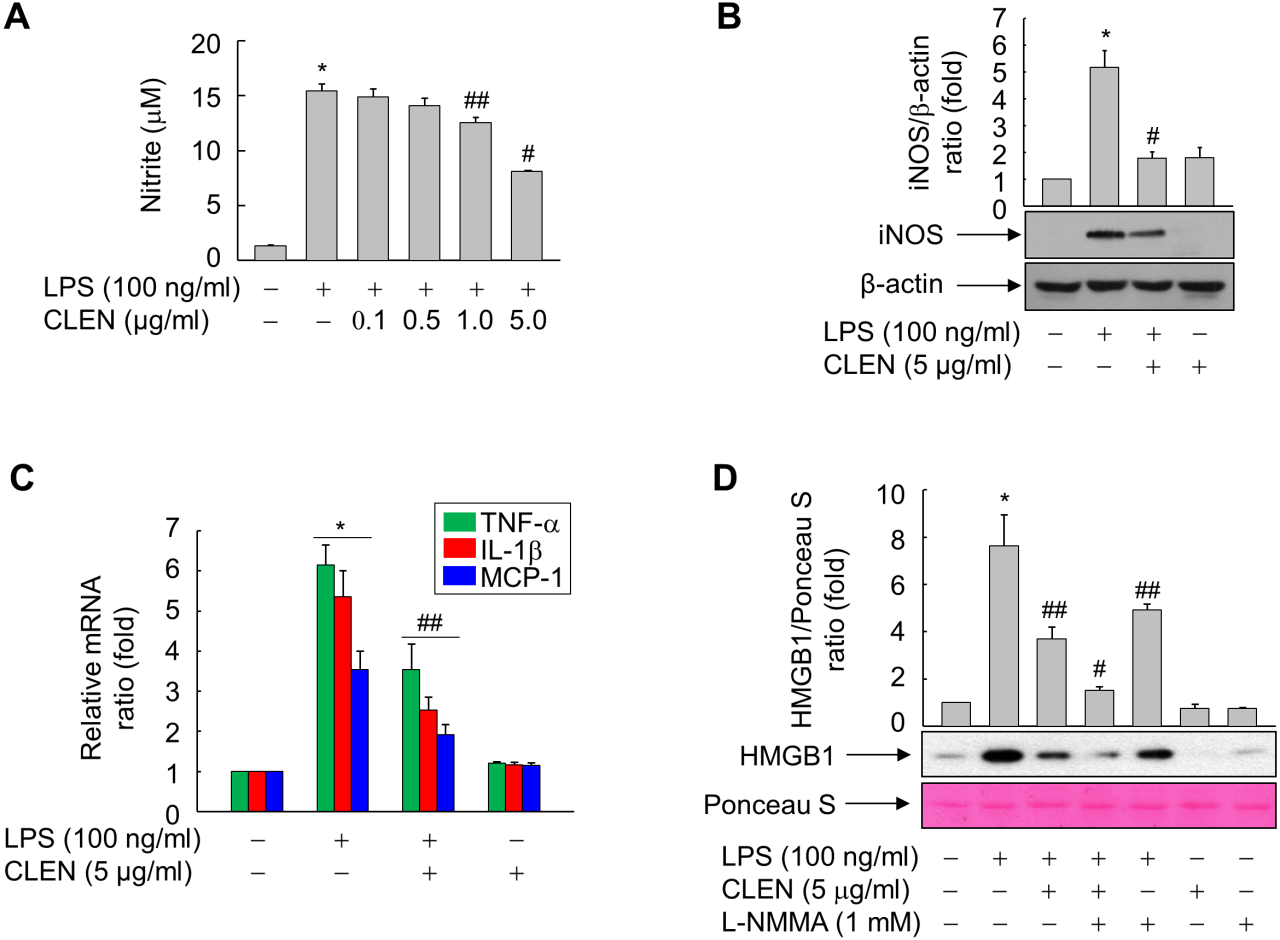
Effect of the *Curcumin longa* extract-loaded nanoemulsion (CLEN) on the production of nitric oxide (NO) and the expression of inducible NO synthase (iNOS), tumor necrosis factor (TNF)- α, interleukin (IL)-6, and monocyte chemotactic protein (MCP)-1 in RAW264.7 cells exposed to lipopolysaccharide (LPS). (A) Cells grown to sub-confluency were maintained in serum-free medium for 24 h and then stimulated with LPS and various concentrations of CLEN for an additional 24 h. Griess reagent was added to equal volumes of conditioned media to measure NO formation. (B and C) Cells maintained in serum-free medium for 24 h were treated for 24 h with LPS in the presence or absence of CLEN. Aliquots of the cell lysates (B) or total RNA (C) were analyzed by immunoblot or real-time PCR, respectively. (D) Cells maintained in serum-free medium for 24 h were pretreated with L-NMMA for 30 min and then treated with LPS in the presence or absence of CLEN. After incubation for 24 h, equal volumes of conditioned media were analyzed by immunoblot with an anti-HMGB1 antibody. Ponceau S staining served as the loading control. In (B) and (D), representative blots from three independent experiments are shown. The band intensities in the three experiments were quantitated by using an image analyzer and the mean ± S.E. ratios of iNOS to *β*-actin (B) or HMGB1 to Ponceau S (D) were plotted (*n* = 3). ^∗^ *p* < 0.01 compared to the untreated cells; ^#^*p* < 0.01, ^##^*p* < 0.05 compared to the cells that were treated with LPS alone.

NO is reported to mediate the LPS-induced release of HMGB1 by RAW264.7 cells (Jiang & Pisetsky, 2006). Therefore, we examined whether CLEN treatment alters the NO production of LPS-treated RAW264.7 cells. While LPS significantly upregulated NO formation, concomitant CLEN treatment reduced it in a dose-dependent manner: the maximal inhibitory effect was observed with 5 µg/ml CLEN (Fig. 3A). Consistent with this result, CLEN treatment also suppressed the LPS-induced expression of iNOS, TNF- α, IL-1 β, and MCP-1 by RAW264.7 cells (Figs. 3B and 3C).

To confirm that CLEN inhibits the LPS-induced release of HMGB1 by suppressing NO formation in RAW264.7 cells, the cells were treated with LPS with and without CLEN in the presence or absence of the iNOS inhibitor ^N^G-monomethyl-L-arginine (L-NMMA). Indeed, L-NMMA significantly potentiated the ability of CLEN to suppress the LPS-induced secretion of HMGB1 (Fig. 3D). This suggests that CLEN inhibits HMGB1 release by suppressing the NO signaling cascade in RAW264.7 cells exposed to LPS.

### Role of mitogen-activated protein (MAP) kinases in CLEN-mediated inhibition of HMGB1 release

**Figure 4:**
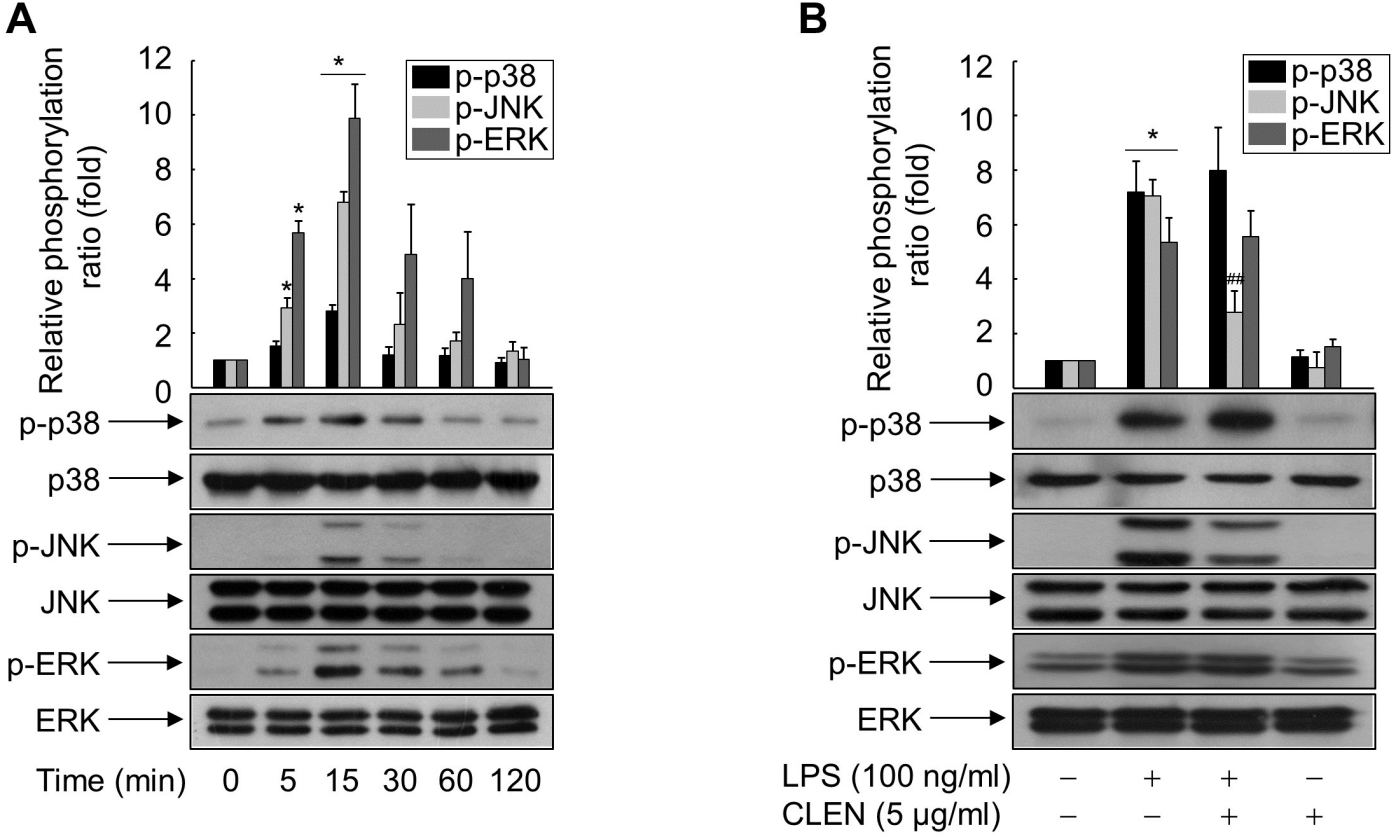
Effect of *Curcumin longa* extract-loaded nanoemulsion (CLEN) on the lipopolysaccharide (LPS)-activated phosphorylation of mitogen-activated protein (MAP) kinases in RAW264.7 cells. (A) RAW264.7 cells grown to sub-confluency were maintained in serum-free medium for 24 h and then exposed to LPS for the indicated times. (B) Cells maintained in serum-free medium for 24 h were treated with or without LPS in the presence or absence of CLEN for 15 min. Aliquots of the cell lysates were immunoblotted with activation-specific antibodies. Parallel immunoblots were analyzed for total kinase levels. Representative blots from three independent experiments are shown. The band intensities in the three experiments were quantitated by using an image analyzer and the mean ± S.E. ratios of phosphorylated kinase to total kinase were plotted (*n* = 3). ^∗^*p* < 0.01 compared to the untreated cells; ^##^*p* < 0.05 compared to the cells that were treated with LPS alone.

To identify the signaling cascade(s) involved in CLEN-mediated inhibition of HMGB1 release by RAW264.7 cells, we analyzed the effect of CLEN on the LPS-induced activation of three MAP kinases, namely, extracellular signal-regulated kinase (ERK), p38, and JNK. As shown in Fig. 4A, LPS activated all three MAP kinases, and their activation peaked at 15 min after stimulation. p38 and ERK activation was sustained for up to 60 min, whereas JNK activation was more transient. Concomitant treatment with CLEN significantly suppressed JNK activation, but not p38 and ERK activation (Fig. 4B). Thus, CLEN appears to inhibit HMGB1 release by suppressing the JNK-mediated signaling cascade.

**Figure 5:**
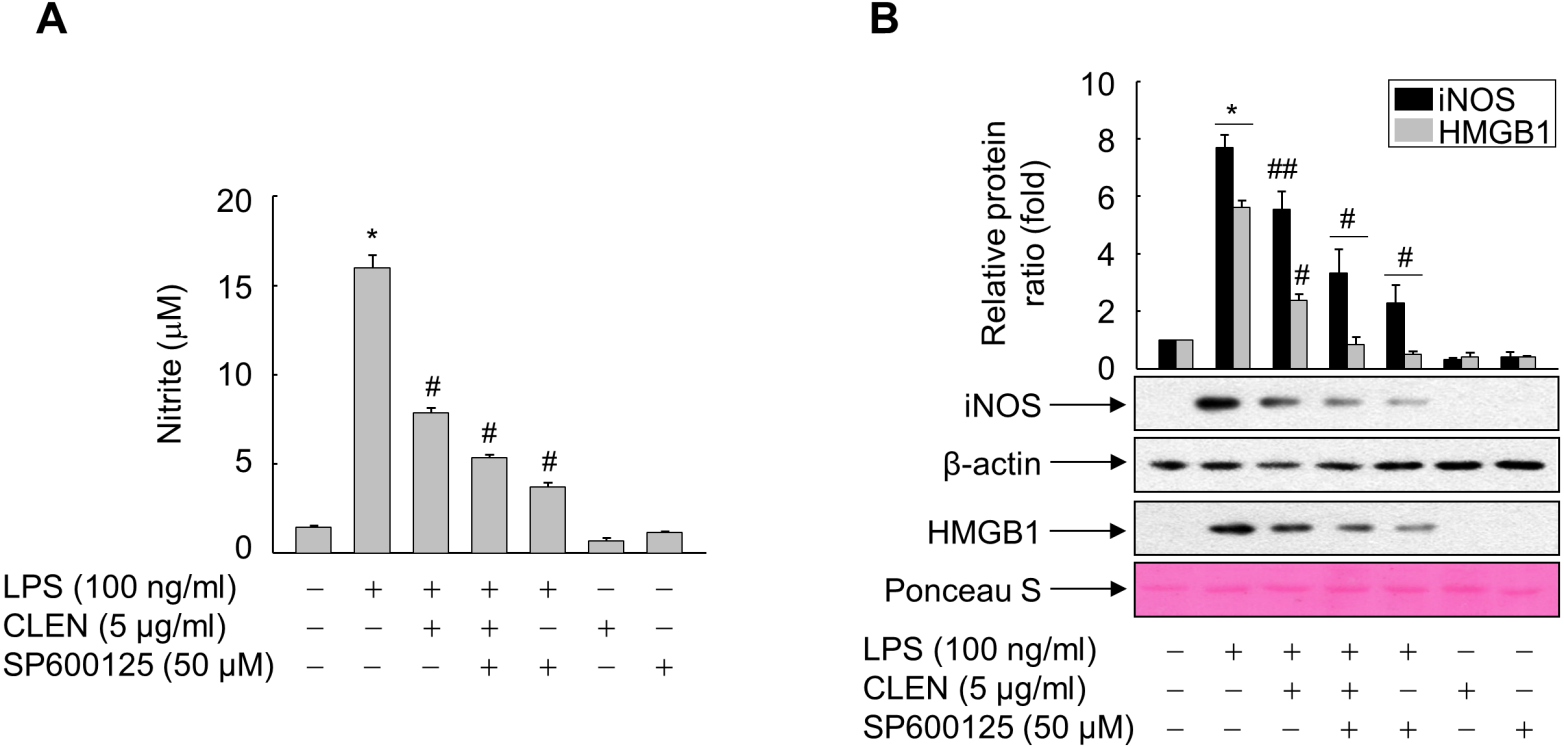
Role of JNK in the ability of *Curcumin longa* extract-loaded nanoemulsion (CLEN) to inhibit the lipopolysaccharide (LPS)-induced release of high-mobility group box 1 (HMGB1). (A and B) RAW264.7 cells grown to sub-confluency were maintained in serum-free medium for 24 h and then exposed to the -Jun N-terminal kinase inhibitor SP600602 for 30 min. The cells were subsequently treated with LPS in the presence or absence of CLEN for 24 h. (A) Equal volumes of conditioned media were subjected to NO formation analysis by using Griess reagent. The results are expressed as mean ± S.E. (*n* = 4). (B) Aliquots of cell lysates and equal volumes of conditioned media were subjected to immunoblot analysis with the indicated antibodies. An antibody specific for *β*-actin and Ponceau S staining served as a loading control. Representative blots from three independent experiments are shown. The band intensities in the three experiments were quantitated by using an image analyzer and the mean ± S.E. ratios of iNOS to *β*-actin (B) or HMGB1 to Ponceau S (D) were plotted (*n* = 3). ^∗^*p* < 0.01 compared to the untreated cells; ^#^*p* < 0.01, ^##^*p* < 0.05 compared to the cells treated with LPS only.

To verify this, we treated LPS-stimulated RAW264.7 cells with CLEN in the presence and absence of SP600125, which is a specific inhibitor of JNK. As shown in Fig. 5, LPS induced the HMGB1 release in a parallel pattern of NO formation and iNOS expression, whereas CLEN significantly suppressed the release of HMGB1, with concomitant reduction in NO level and iNOS expression. This CLEN-mediated inhibitory activity is further potentiated in the presence of SP600125, suggesting that CLEN inhibits the LPS-induced release of HMGB1 by suppressing the JNK-mediated NO signaling cascade.

### Effect of CLEN on endotoxin-induced death in a murine model of septicemia

**Figure 6:**
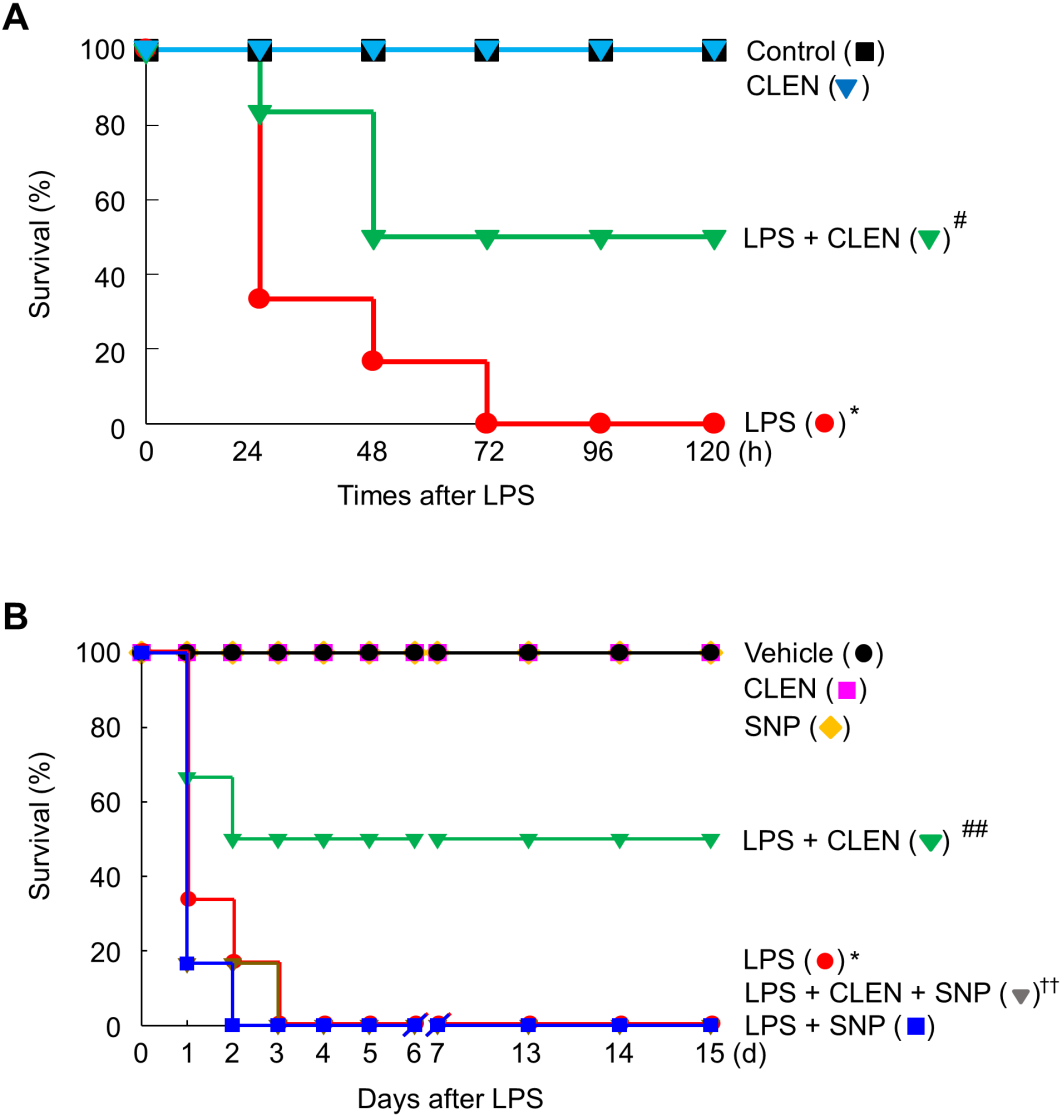
Effect of *Curcumin longa* extract-loaded nanoemulsion (CLEN) injection on endotoxin-induced mortality of mice. Male (A) or female (B) BALB/c mice (*n* = 6 per group) were injected with a single dose of CLEN (50 mg/kg, i.p.) in the presence or absence of a lethal infusion of endotoxin (LPS, 10 mg/kg, i.p.) and/or SNP (2.5 mg/kg, i.p.). Survival was monitored daily for up to 15 days. Survival was plotted. ^∗^*p* < 0.01, ^∗∗^*p* < 0.05 compared to the untreated mice; ^#^*p* < 0.01, ^##^*p* < 0.01 compared to the mice that were given LPS only; ^††^*p* < 0.05 compared to the LPS plus CLEN-treated mice.

Our findings led us to experiments in mice with endotoxemia, which is a model of systemic inflammation. These mice were used to examine whether CLEN treatment can protect the mice from death by suppressing HMGB1 release into the circulation during endotoxemia. Thus, the male or female mice were injected with LPS in the presence or absence of CLEN and/or SNP to verify gender difference. LPS alone led to the death of all mice (100%) within 72 h of the injection. However, concomitant i.p. treatment with CLEN reduced the mortality rate to 50%. This protective effect of CLEN was not observed in mice treated with SNP, indicating that CLEN-mediated blockade of NO signaling is critical in LPS-induced lethality. Interestingly, there were no gender difference in the inhibitory effects of CLEN against LPS-induced lethality (Fig. 6). Moreover, all of the CLEN-treated animals who survived continued to survive up to 15 days after LPS challenge; there were no late deaths. This suggests that CLEN treatment protected the mice from lethal endotoxemia.

**Figure 7:**
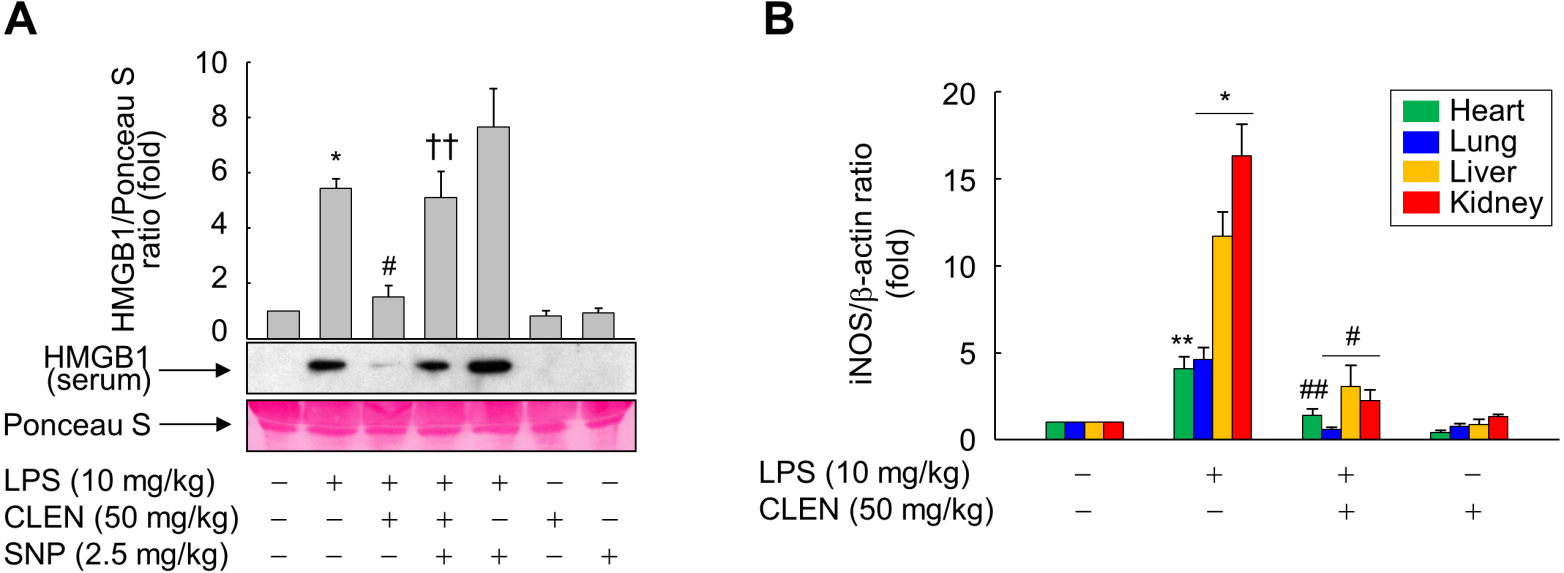
Effect of *Curcumin longa* extract-loaded nanoemulsion (CLEN) on the circulating high-mobility group box 1 (HMGB1) levels and tissue expression of inducible nitric oxide synthase (iNOS) in endotoxemic mice. (A and B) BALB/c mice (*n* = 3 per group) were injected with a single dose of CLEN (50 mg/kg, i.p.) in the oresence or absence of a lethal infusion of endotoxin (LPS, 10 mg/kg, i.p.) and/or SNP (2.5 mg/kg, i.p.). (A) The circulating HMGB1 levels were measured by immunoblot analysis of sera. (B) The iNOS expression in various tissues collected 18 h post-LPS injection was also determined by immunoblot analysis. Ponceau S staining and *β*-actin served as loading controls in (A) and (B), respectively. In (A), the results are expressed as mean ± S.E. (*n* = 3). In (B), The band intensities of blots from three independent experiments were quantitated by using an image analyzer and the mean ± S.E. ratios of iNOS to *β*-actin were plotted (*n* = 3). ^∗^*p* < 0.01, ^∗∗^*p* < 0.05 compared to the untreated mice; ^#^*p* < 0.01, ^##^*p* < 0.05 compared to the mice that were treated with LPS only; ^††^*p* < 0.05 compared to the LPS plus CLEN-treated mice.

Since HMGB1 exacerbates inflammatory responses during LPS-induced pathogenic endotoxemia (Wang et al., 1999), we examined the effect of CLEN on the circulating levels of HMGB1. While the circulating levels of HMGB1 rose following LPS challenge, concomitant CLEN treatment significantly reduced these levels (Fig. 7A). We also found that concomitant CLEN treatment markedly inhibited the LPS-induced iNOS expression in various tissues, including the heart, lung, liver, and kidney (Fig. 7B). These data suggest that CLEN protected the mice from endotoxin-induced death by blocking NO signaling, thereby suppressing the release of HMGB1 into the circulation.

## Discussion

An increasing body of evidence indicates that secreted extracellular HMGB1 plays an important role in the pathogenesis of inflammation-related diseases. The next step is to determine the mechanisms involved and to develop therapeutic strategies that will block the secretion of HMGB1 into the extracellular fluid. The present study showed that when LPS-stimulated murine macrophages were treated with a herbal extract nanoemulsion, it significantly inhibited their release of HMGB1 into the extracellular fluid. Moreover, L-NMMA, specific inhibitor for iNOS, further potentiated the ability of CLEN to inhibit LPS-induced HMGB1 release. Thus, CLEN appears to suppress LPS-induced HMGB1 secretion by inhibiting NO signaling. This is supported by the findings of Jiang and Pisetsky: they found that NO signaling, as well as interferon-alpha, associated with HMGB1 release (Jiang & Pisetsky, 2006). Thus, our study adds to the body of work that has shown that extracts and compounds from other herbs can block HMGB1 release (Wu et al., 2015; Zhang et al., 2012; Li et al., 2011; Sakamoto et al., 2001). Additional studies are needed to elucidate further whether and how these agents, and CLEN, protect from HMGB1-associated inflammatory disorders such as septicemia.

Although LPS activated three MAP kinases in murine macrophages, only JNK was targeted by CLEN: while LPS activated p38, ERK, and JNK, CLEN only attenuated the LPS-induced rise of JNK activation. In line with this finding, SP600125, an inhibitor of JNK, potentiated the ability of CLEN to inhibit the LPS-induced release of HMGB1 by macrophages. This observation is consistent with the previous study that intravenous administration of SP600125 in ischemia-reperfusion model rats not only suppressed JNK expression in the heart tissue, it also significantly reduced the serum HMGB1 levels (Zhai et al., 2012). A similar relationship between HMGB1 and JNK was observed when normal human bronchial epithelial cells were cultured with HMGB1 in the presence or absence of SP600125: the HMGB1-induced inflammatory response was suppressed by SP600125 (Wu et al., 2013). By contrast, Wang et al. (2015) reported that the MAP kinase p38 mediates the inhibitory effect of ketamine on the LPS-induced secretion of HMGB1 in murine macrophages by upregulating the expression of heme oxygenase-1. Other studies have also shown that several natural products, namely, Dachengqi decoction (a traditional Chinese medicine formula), glycyrrhizin (from licorice root), and the tetrahydroisoquinoline alkaloid THI-28, inhibit HMGB1 release in cellular and animal models of endotoxemia and severe acute pancreatitis by activating p38 signaling (Kim, Kim & Chang, 2015; Chen et al., 2015; Kim et al., 2013). Thus, it remains unclear which MAP kinase associates with the inhibition of HMGB1 release; the observed discrepancies may reflect the nature of the natural products being tested. In any case, the present study clearly showed that the JNK signal is involved in the CLEN-mediated inhibition of HMGB1 release by macrophages.

Consistent with our *in vitro* finding in murine macrophages, administration of CLEN significantly improved the survival of mice with LPS-induced endotoxemia. Moreover, this improved survival rate associated with reduced circulating HMGB1 levels. This suggests that CLEN protects from endotoxemia by targeting HMGB1. Several previous studies have also found that *C. longa* extracts or compounds regulate inflammatory reactions. First, Sengupta et al. showed that an aqueous fraction of *C. longa* suppresses the release of prostaglandin E2 and interleukin-12 by LPS-stimulated normal mouse splenocytes (Sengupta, Sharma & Chakraborty, 2011). Second, Somchit et al. showed that a curcumanoid from *C. longa* inhibits the NO signaling pathway in inflamed human intestinal CaCo cells (Somchit et al., 2014). Third, Xu et al. showed that terpenoids from a *C. longa* extract inhibited the NO production by murine LPS-primed microglial BV2 cells (Xu et al., 2015). Thus, *C. longa* may block the LPS-triggered HMGB1 release of macrophages by inhibiting NO-associated cellular signaling.

## Conclusions

We demonstrated here that CLEN suppressed the LPS-induced release of HMGB1 by macrophages, and that this effect was due to CLEN inhibition of JNK-mediated NO signaling. We also showed that this inhibitory activity of CLEN was associated with reduced mortality in endotoxemic mice. Taken together, our findings indicate that natural compounds, including those of the herb *C. longa*, that may prevent HMGB1 production and/or activity may aid the treatment of inflammation-associated disorders such as endotoxemia.

##  Supplemental Information

10.7717/peerj.3808/supp-1 Supplemental Information 1 Raw data for Western blots Uncropped blots for each figure.

10.7717/peerj.3808/supp-2 Supplemental Information 2 Raw data for statistics Raw and statistical data of MTT assay for Fig. 2A.

10.7717/peerj.3808/supp-3 Supplemental Information 3 Raw data for statistics Raw and statistical data for the ratios of HMGB1 to Ponceau S of Fig. 2B.

10.7717/peerj.3808/supp-4 Supplemental Information 4 Raw data for statistics Raw and statistical data of nitrite measured by Griess reagent in condition media for Fig. 3A.

10.7717/peerj.3808/supp-5 Supplemental Information 5 Raw data for statistics Raw and statistical data for the ratios of iNOS to b -actin of Fig. 3B.

10.7717/peerj.3808/supp-6 Supplemental Information 6 Raw data for statistics Raw and statistical data of real-time pcr for Fig. 3C.

10.7717/peerj.3808/supp-7 Supplemental Information 7 Raw data for statistics Raw and statistical data for the ratios of HMGB1 to Ponceau S of Fig. 3D.

10.7717/peerj.3808/supp-8 Supplemental Information 8 Raw data for statistics Raw and statistical data for the ratios of phosphorylation to each control of Fig. 4A.

10.7717/peerj.3808/supp-9 Supplemental Information 9 Raw data for statistics Raw and statistical data for the ratios of phosphorylation to each control of Fig. 4B.

10.7717/peerj.3808/supp-10 Supplemental Information 10 Raw data for statistics Raw and statistical data of nitrite measured by Griess reagent in condition media for Fig. 5A.

10.7717/peerj.3808/supp-11 Supplemental Information 11 Raw data for statistics Raw and statistical data for the ratios of iNOS and HMGB1 to each control of Fig. 5B.

10.7717/peerj.3808/supp-12 Supplemental Information 12 Raw data for statistics Raw and statistical data for survival rates (%) of mice challenged by LPS and CLEN of Fig. 6A.

10.7717/peerj.3808/supp-13 Supplemental Information 13 Raw data for statistics Raw and statistical data for survival rates (%) of mice challenged by LPS, CLEN and SNP of Fig. 6B.

10.7717/peerj.3808/supp-14 Supplemental Information 14 Raw data for statistics Raw and statistical data for the ratios of HMGB1 to Ponceau S of Fig. 7A.

10.7717/peerj.3808/supp-15 Supplemental Information 15 Raw data for statistics Raw and statistical data for the ratios of iNOS to b -actin of Fig. 7B.

## Additional Information and Declarations

### Data Availability

The following information was supplied regarding data availability: The raw data is uploaded as Supplemental Files.
