# Supplementary figures and images for: Curcumin longa extract-loaded nanoemulsion improves the survival of endotoxemic mice by inhibiting nitric oxide-dependent HMGB1 release
Source: PeerJ. 2017 Sep 14;5:e3808. doi: 10.7717/peerj.3808 (PMC5600948; doi:10.7717/peerj.3808)

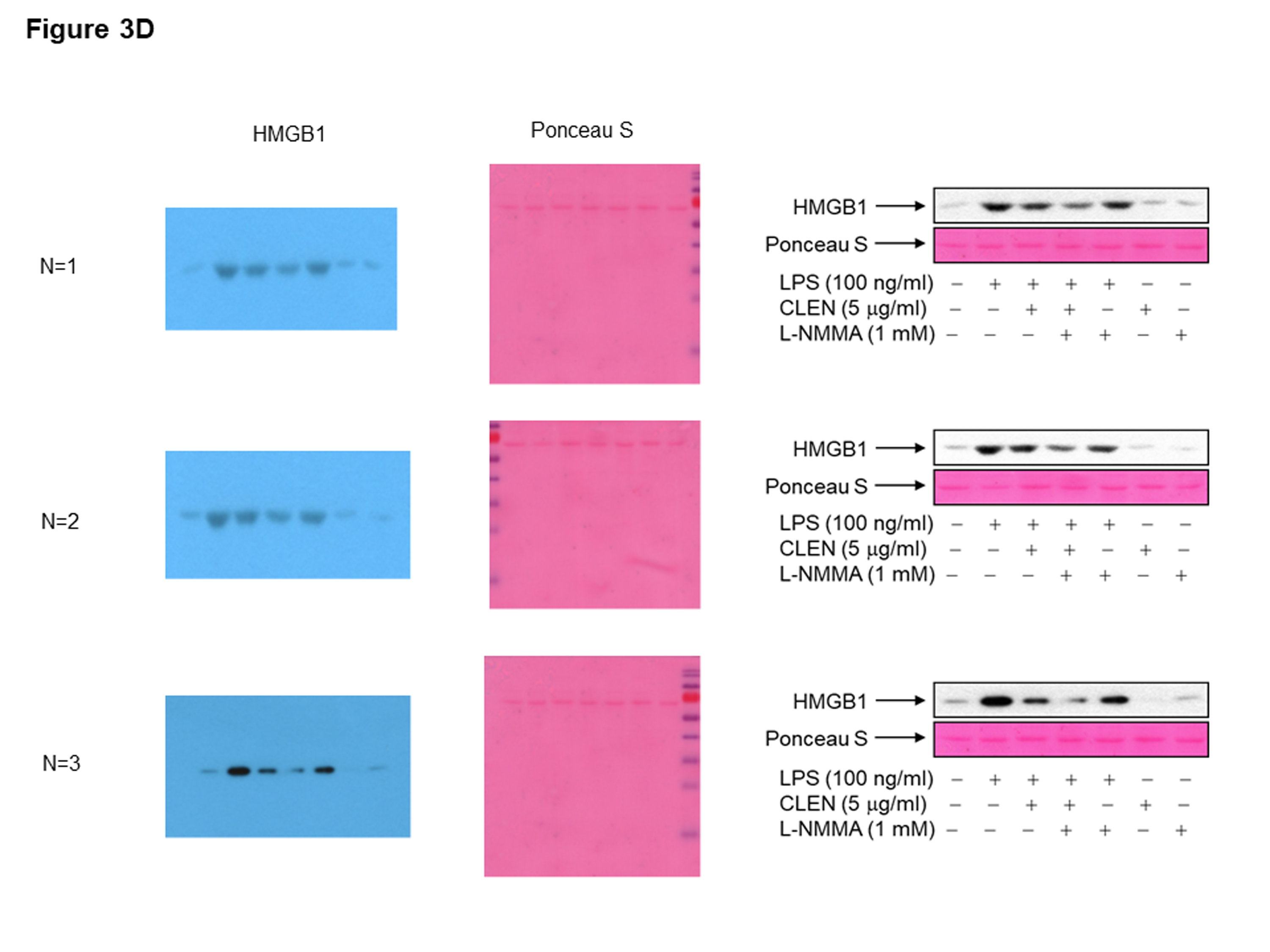

Supplement: Supplemental Information 1 — Uncropped blots for each figure. [file peerj-05-3808-s001.zip › WB raw data for Figure 3D.PNG]

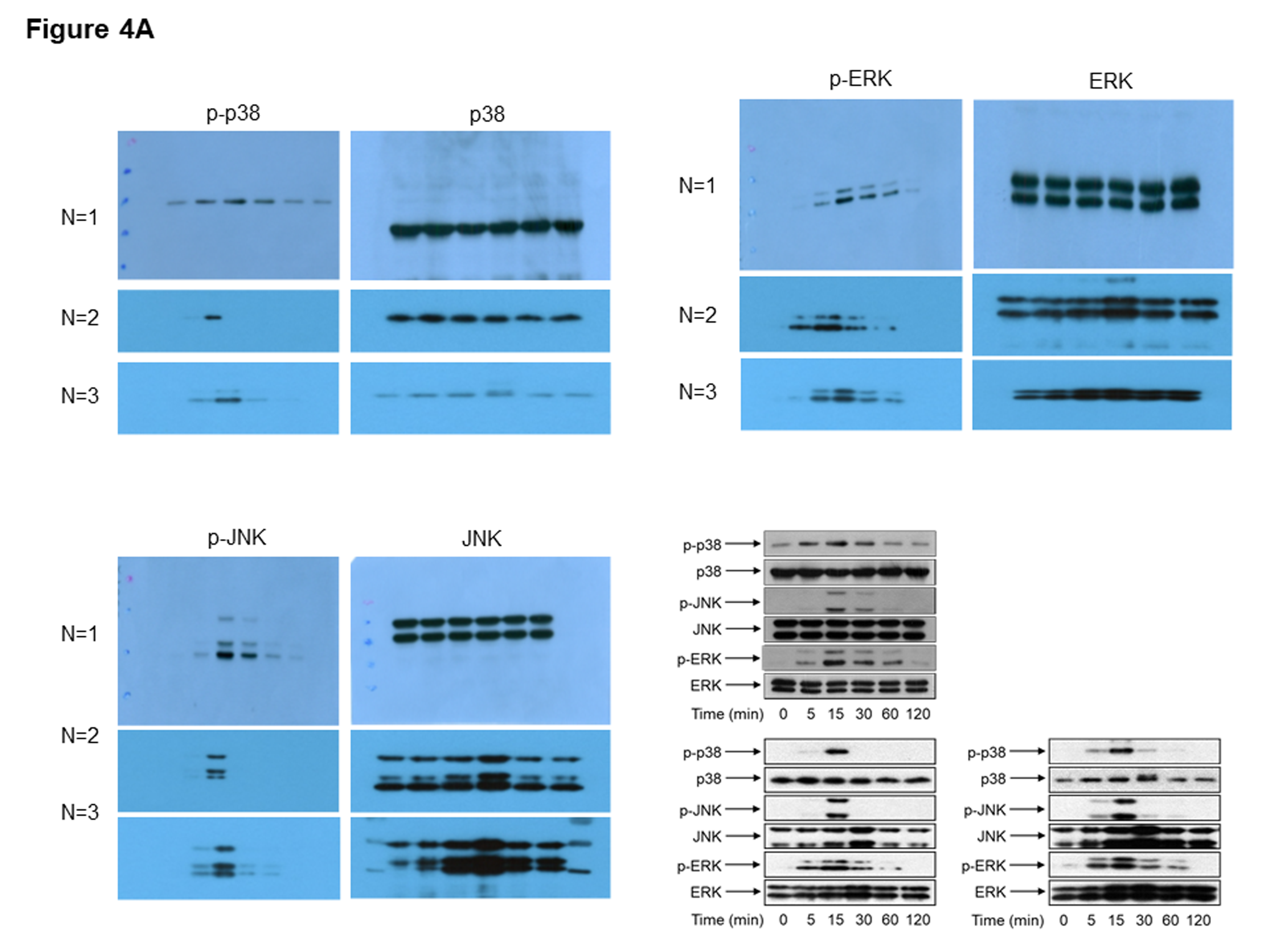

Supplement: Supplemental Information 1 — Uncropped blots for each figure. [file peerj-05-3808-s001.zip › WB raw data for Figure 4A.PNG]

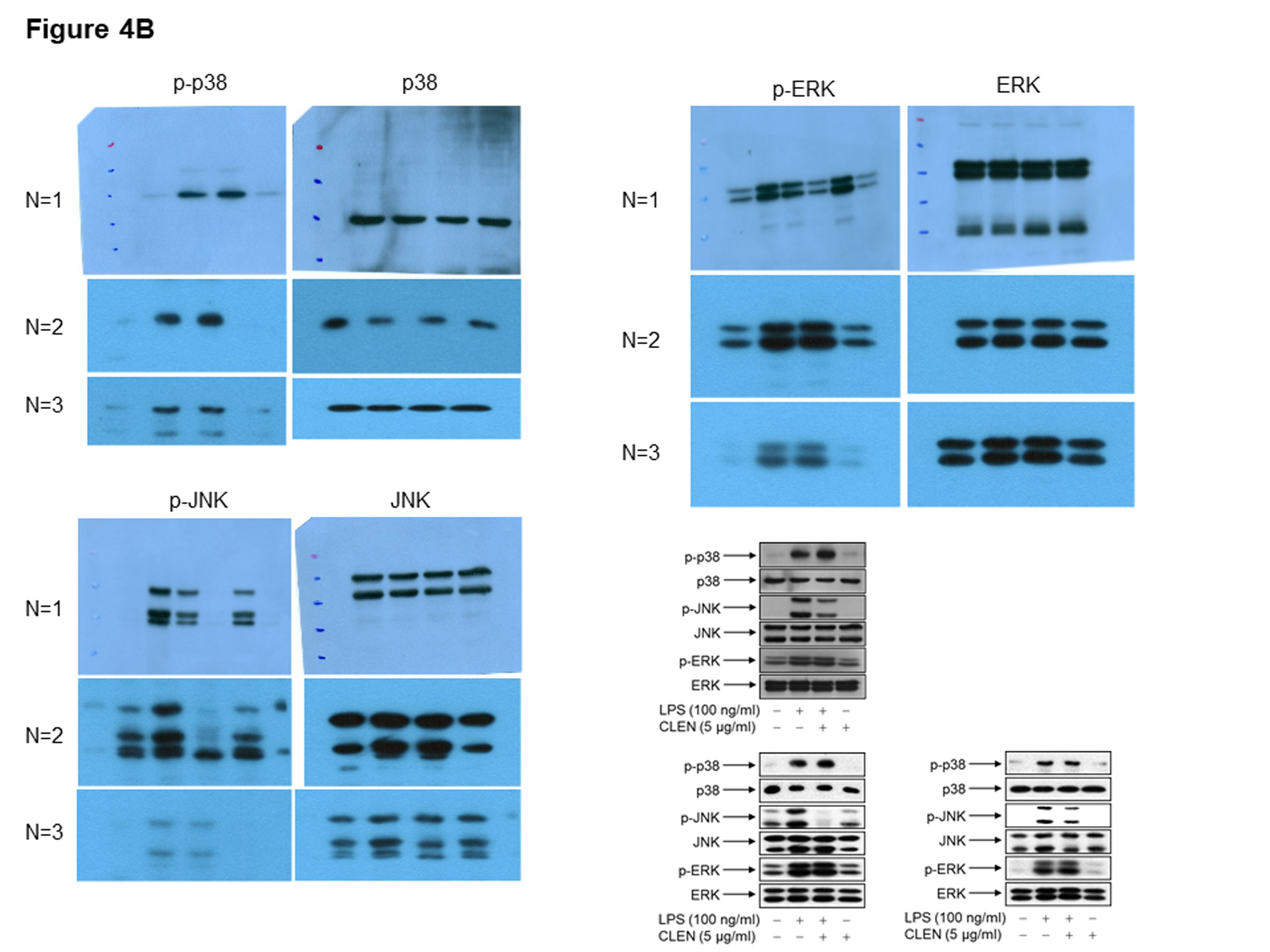

Supplement: Supplemental Information 1 — Uncropped blots for each figure. [file peerj-05-3808-s001.zip › WB raw data for Figure 4B.PNG]

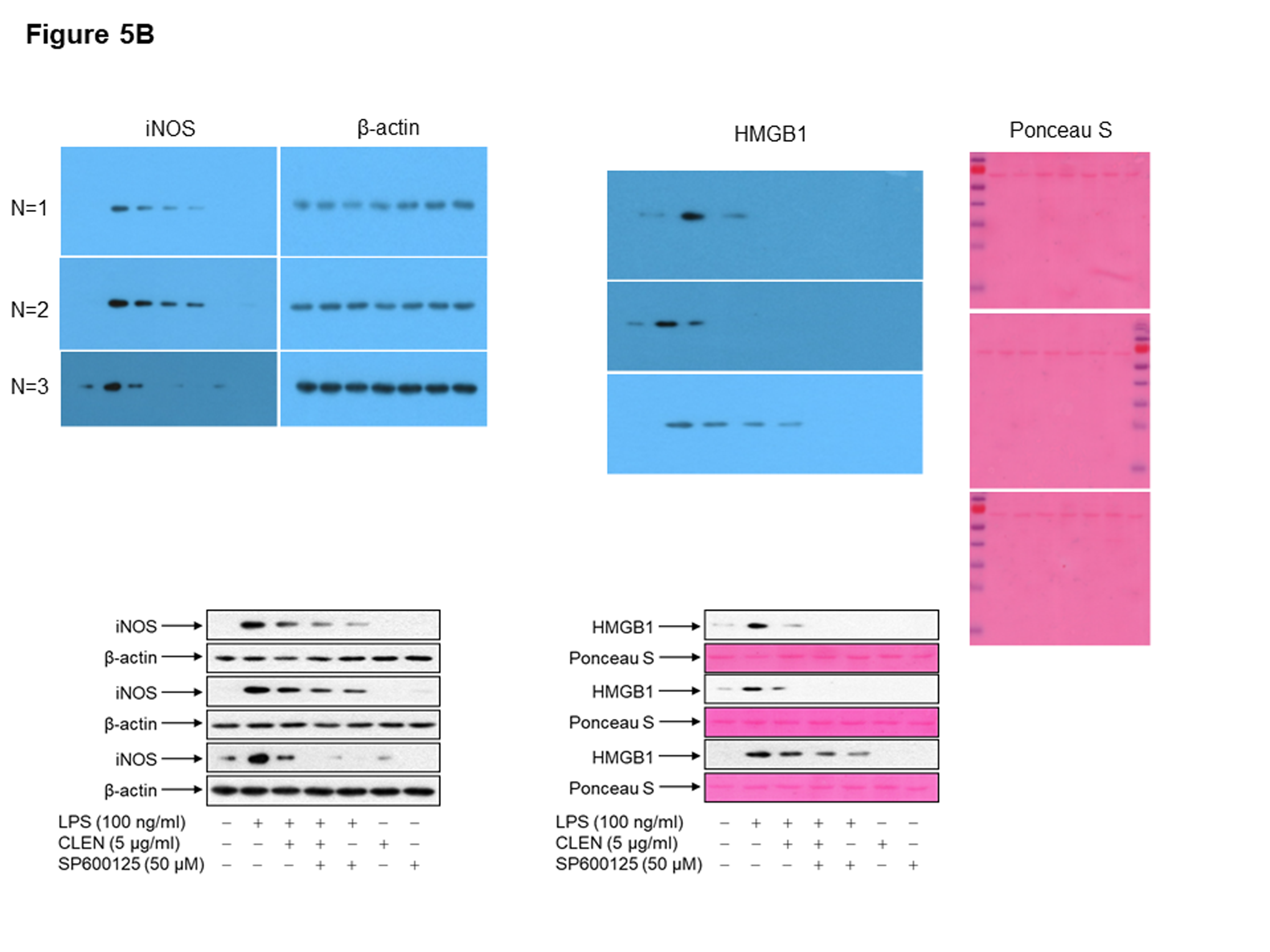

Supplement: Supplemental Information 1 — Uncropped blots for each figure. [file peerj-05-3808-s001.zip › WB raw data for Figure 5B.PNG]

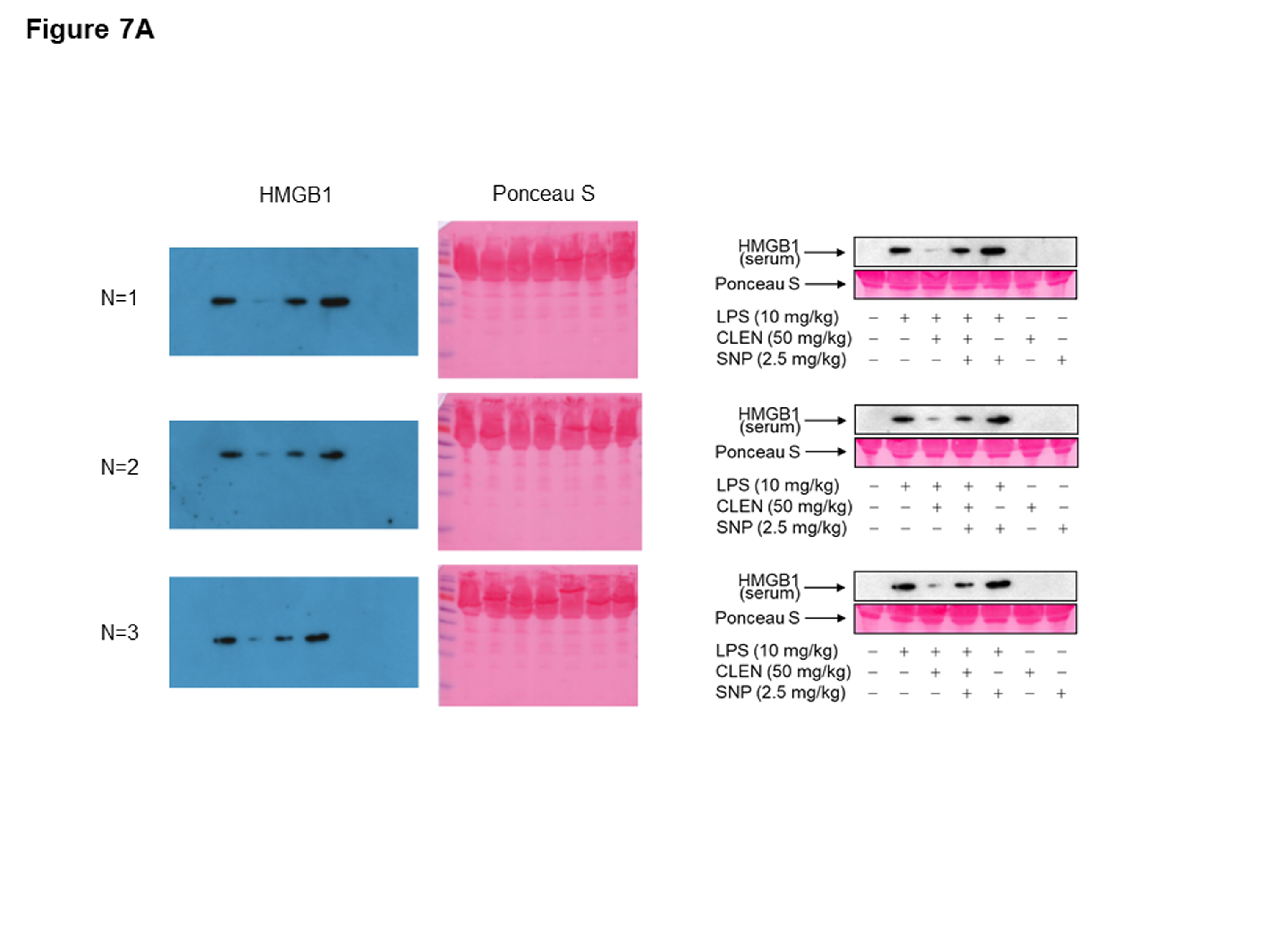

Supplement: Supplemental Information 1 — Uncropped blots for each figure. [file peerj-05-3808-s001.zip › WB raw data for Figure 7A.PNG]

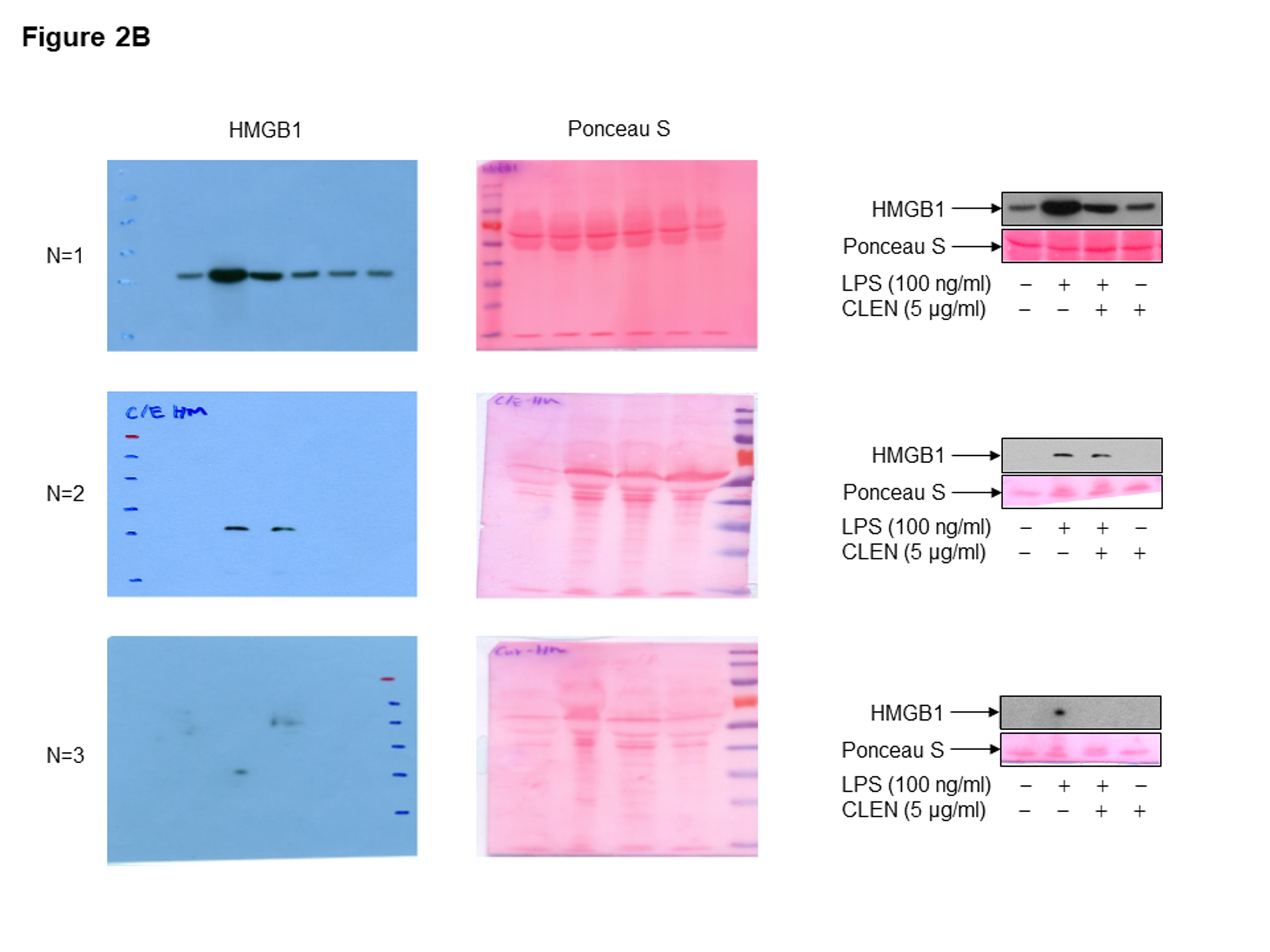

Supplement: Supplemental Information 1 — Uncropped blots for each figure. [file peerj-05-3808-s001.zip › WB raw data for Figure 2B.PNG]

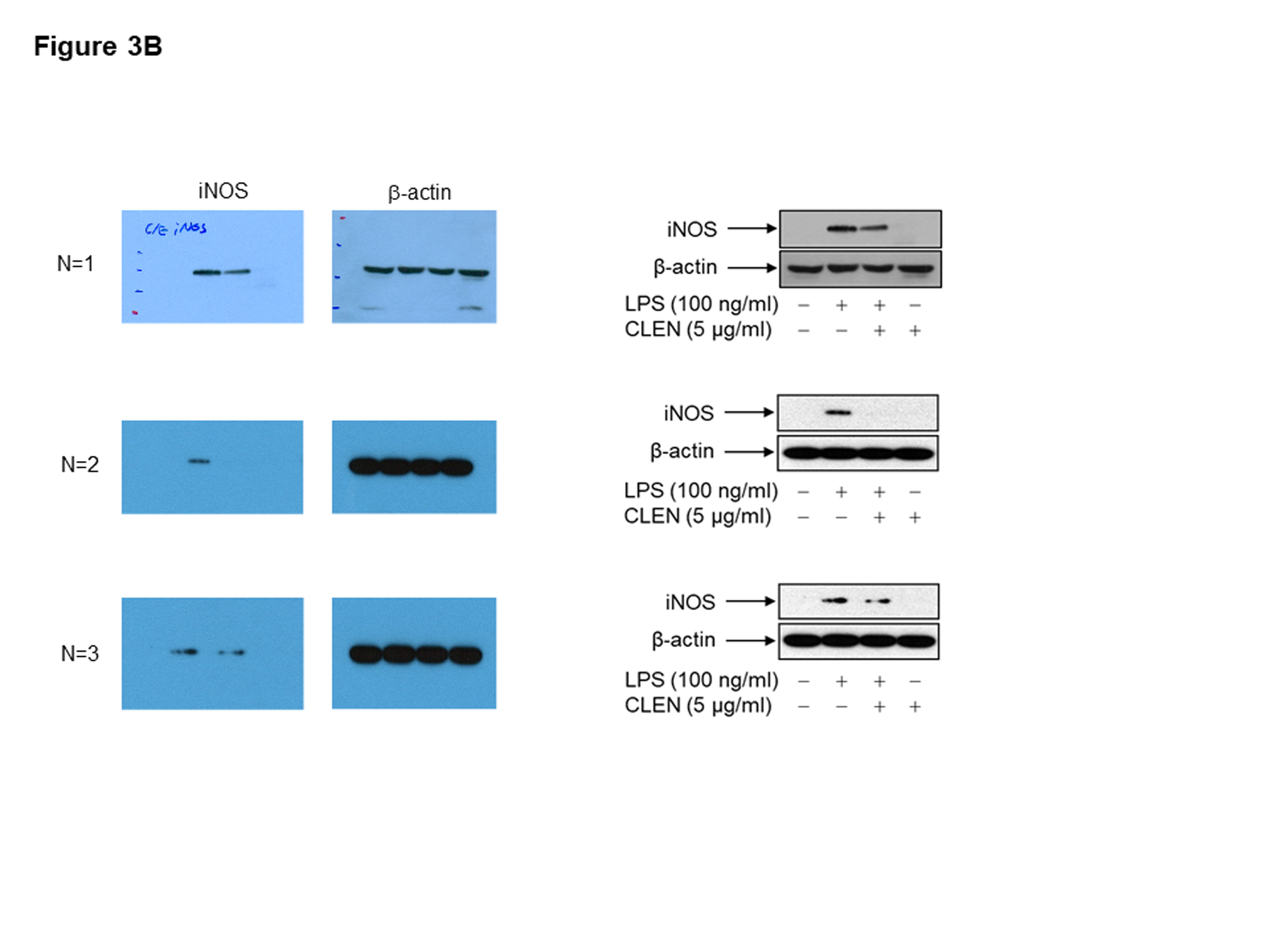

Supplement: Supplemental Information 1 — Uncropped blots for each figure. [file peerj-05-3808-s001.zip › WB raw data for Figure 3B.PNG]
